# Minimally Invasive Interventions in Obstructive and Inflammatory Salivary Gland Diseases: Local Anesthesia Based Pain Management, Stratification of Invasiveness, and Patients’ Perceptions

**DOI:** 10.3390/jcm14061797

**Published:** 2025-03-07

**Authors:** Mirco Schapher, Maximilian Traxdorf, Heinrich Iro, Michael Koch

**Affiliations:** 1 Department of Otolaryngology, Head and Neck Surgery, Friedrich-Alexander-Universität Erlangen-Nürnberg, Universitätsklinikum Erlangen, Waldstrasse 1, 91054 Erlangen, Germanymichael.koch@uk-erlangen.de (M.K.); 2 Department of Otolaryngology, Head and Neck Surgery, Klinikum Nuernberg, Paracelsus Medical University, Prof.-Ernst-Nathan-Strasse 1, 90419 Nuremberg, Germany

**Keywords:** inflammatory and obstructive salivary gland diseases, sialadenitis, sialolithiasis, sialodochitis, sialendoscopy, minimal invasive treatment, local anesthesia, pain management, patients’ perceptions

## Abstract

**Objectives:** Since the peri- and intraoperative management of patients with inflammatory and obstructive sialadenitis (IOS) differs significantly between treating centers worldwide, we investigated whether these patients can be treated successfully, resource-savingly and with high patient satisfaction using minimally invasive procedures under local anesthesia (LA). **Methods:** We developed a comprehensive, stratified routine anesthesia and pain management protocol based on our proposed classification of invasiveness (grade 1–4), for almost all available IOS treatment procedures. We included 377 patients with 470 LA-conducted interventions in our study and evaluated their perceptions during and after the treatment. **Results:** The protocol was applied to all 377 study participants for all 470 interventions. The mean grade of invasiveness was 2.49 ± 1.31, with a mean procedure duration of 30 ± 20 min. We found a significant positive association between invasiveness levels and procedure duration (*p* = 0.001) or pain directly after surgery (*p* = 0.004). Patients rated the procedures as ”well acceptable” or better in a large majority (88.1–97%) regarding the administration and potency of LA, procedure duration, and pain during and directly after surgery. In total, 96.4% of patients would have the treatment repeated under the same conditions. **Conclusions:** The proposed anesthesia and pain management regimen, respecting invasiveness levels, enables IOS patients to undergo treatment under LA with high success rates, serving as a potential guide for performing physicians.

## 1. Introduction

The most relevant causes of obstructive sialadenopathies affecting the major salivary glands in adults are sialolithiasis in 40–85% [1,2,3,4], duct stenosis in 5–25% [5], and inflammatory changes in the duct system in 5–15% [2], including sialodochitis. In children, the most common causes for sialadenitis are chronic recurrent juvenile parotitis (CRJP) and mumps [6,7,8].

Diagnostic and therapeutic options in inflammatory and obstructive sialadenitis (IOS), particularly regarding sialolithiasis and stenosis, have changed tremendously over the last three decades [9,10,11,12,13,14,15,16,17]. The development of sialendoscopy (SE) was a milestone because it allowed the direct visualization of the causative pathology independent of other imaging techniques [9,10,11,12]. SE is used in the implementation of intraductal/intracorporeal shock wave lithotripsy (ISWL) [13,14,15], various methods of transoral duct surgery (TDS) [16,17], duct recanalization and stenting [5,18,19], endoscopy-assisted transoral/transcutaneous operation techniques [20,21], and in monitoring and supplementing extracorporeal shock wave lithotripsy (ESWL) [22,23]. The usage of all these techniques has resulted in a gland preservation rate of over 95% today [4,17,24].

Treatment routines have been elaborated worldwide with growing experience for the most common pathological conditions, particularly for sialolithiasis [2,3,25,26,27,28]. Although different multimodal therapeutic concepts and algorithms have been published [1,2,3,24,27,28,29,30,31,32], even recognized and proven techniques are not yet standardized or classified into different levels of invasiveness. Therefore, systematic studies on the appropriate form of anesthesia and pain management are not available, and the results and experiences are difficult to compare. While “less invasive interventions” can be performed under local anesthesia (LA) [29,30,33,34,35], other experiences published so far differ considerably. For example, SE-assisted duct irrigation (with isotone saline solution ± cortisone) is recognized as an effective therapy to treat patients with CRJP, sialodochitis or Sjoegren’s syndrome [2,7,8,24,29,33,35,36,37]. In children with CRJP, interventions are usually performed under general anesthesia (GA) or deep sedation in over 90 per cent of cases [7,8,36,37]. However, in a small study, a mere irrigation (without SE) was possible and beneficial without any kind of anesthesia [33]. In adults, this intervention could be carried out with or even without LA successfully in all cases [29,35].

Next, TDS in the submandibular gland varies considerably, starting from a less invasive papillotomy up to much more invasive extended duct surgery including a submandibulotomy. Extended submandibular TDS was performed under GA in more than 95% of cases [38,39,40,41], but other centers have also successfully performed it under LA in 93–98% of cases [16,17,42,43].

The same is true for intraductal SE-assisted interventions, such as ISWL or the treatment of stenosis. Depending on the performing center, these interventions are carried out in 61–100% under GA [44,45,46,47,48] or in 70–100% of cases under LA [13,14,15,24,49,50,51,52,53,54,55]. SE-assisted transcutaneous parotid gland surgery is performed under GA in the majority of centers [21,56,57,58], but has also been conducted successfully under LA in 89–100% of patients elsewhere [20,59,60]. ESWL is conducted under LA or without any kind of anesthesia by nearly all centers in adult patients [54,61,62,63,64,65,66,67,68], while the treatment of children mostly required sedation or GA [62].

We perform more than 98% of all interventions under LA in our department [3,4,5,13,14,15,16,17,18,19,22,26,42,49,54,69,70,71,72,73]. LA eliminates the risks of GA (facilitating therapy even in multimorbid patients), saves operating room capacities for procedures that inevitably require GA, shortens the inpatient stay duration, and reduces treatment costs [74,75,76].

To assess the acceptance of minimally invasive interventions in IOS patients performed under LA, we categorized the various interventions according to their invasiveness. Adapted to this classification, we designed a systematic and graduated LA-based pain management protocol. The latter was applied in all interventions of all patients included in this study, and we systematically recorded and analyzed the patients’ perceptions of each individual procedure during and after the treatment.

## 2. Materials and Methods

### 2.1. Data Acquisition and Ethics

The data were obtained at an academic tertiary referral center specializing in salivary gland diseases (Department of Otolaryngology, Head and Neck Surgery, University of Erlangen-Nuremberg, Erlangen, Germany). We received informed consent from each patient involved in this study, respecting the university’s general contract conditions and the World Medical Association’s Declaration of Helsinki. Approval was obtained from the local institutional review board of the University of Erlangen-Nuremberg (186_19 Bc).

### 2.2. Study Population

**Figure 1:**
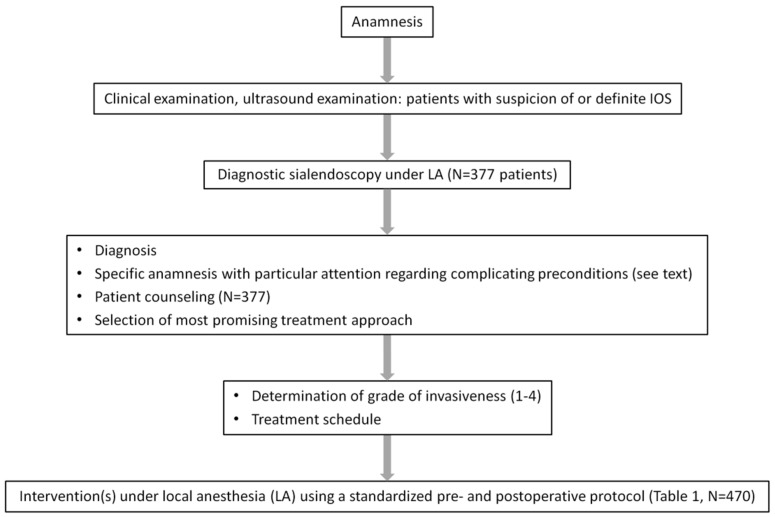
Study population, workflow, and treatment in patients with inflammatory and obstructive sialadenitis (IOS). IOS: Inflammatory and obstructive sialadenitis; LA: Local anesthesia.

Patients presenting with IOS who were diagnosed at our department between February 2019 and May 2020 and agreed to participate formed our study cohort (Figure 1). Patients who were treated routinely under GA (combined endoscopic–transcutaneous approach in the parotid gland [PG], children under 12 years of age) were not considered in this study. All other treatments were planned and performed under LA. For better understanding and clarity, we analyzed each hospital stay as a whole, categorized it according to the intervention step with the highest grade of invasiveness performed during the stay, and refer to it as “intervention” or “procedure”.

### 2.3. Diagnostic Procedures and Patient Counseling

The preoperative measures included a thorough clinical and ultrasound (US) examination in each patient, using a high-end US device (Acuson Sequoia, 10L4 transducer, Siemens Healthineers, Erlangen, Germany) with gland stimulation [77]. All US examinations were conducted by either the first or the senior author of this study, who both are certified and experienced ultrasound instructors (DEGUM, German Society of Ultrasound in Medicine). In case of suspected or manifest IOS, a video recorded diagnostic SE was performed using sialendoscopes as described previously [3,5,13,14,15,16,17,22,32,49,50,55,69,71]. Afterwards, the most promising treatment approach was selected for each patient, according to our algorithms described before [2,3,17].

Patients with potentially complicating preconditions (high level of anxiety; unfavorable anatomy of the floor of the mouth (FOM); restricted mouth opening; presence of a pronounced gag reflex; tongue hyperplasia, presence of OSAS with limited possibility to retract the tongue) were carefully assessed and counseled (Figure 1).

### 2.4. Grade of Invasiveness, Local Anesthesia, Pre- and Postoperative Medication

We classified every intervention, according to its invasiveness, into one of four grades. In addition to the surgical procedure itself, the expected duration and the expected need for anesthetic medication were also taken into account. For each category, we established a standardized protocol, containing precise instructions for local anesthesia and for pre-, intra- and postoperative medications (Table 1).

Every patient received mucosa anesthesia (lidocaine spray 2%, AstraZeneca GmbH, Wedel, Germany). Subsequently, adjusted to the grade of invasiveness, intraductal anesthesia was applied by instillation into the duct (ultracain: articaine hydrochloride 2% with 0.006 mg/mL epinephrine, Septodont GmbH, Niederkassel, Germany). Furthermore, we injected another 2–10 mL of ultracain, adjusted to the grade of invasiveness, into the adjacent area of the treated duct or gland (FOM, cheek). If ESWL was performed, 5 mL of ultracain was injected into the FOM, adjacent to the mandible, and 2–4 mL was additionally injected transcutaneously directly into the stone bed within the gland (Table 1).

Patients were treated postoperatively as described (Table 1). Supplementary and regular oral rinses (Salviathymol; Meda Pharma Ltd., Bad Homburg, Germany) as well as regular daily gland massages using ascorbic acid or sialagogues were recommended.

### 2.5. Treatment Procedures

Various endoscopes (0.8–1.6 mm) were used for diagnostic SE, therapeutic irrigation, or interventional SE, as described elsewhere [13,14,15,16,17,22,73]. TDS was performed in the submandibular gland (SMG) only as described before [16,17]. The level of invasiveness in TDS ranged from grade 2 (papillotomy, distal third of the duct) up to grade 4 (extended TDS: entire length of the duct beyond the hilum and into the gland tissue, submandibulotomy), including the creation of a neo-ostium [16,17]. In this study, we used stents for PG treatment exclusively [2,3,19] (Table 1). Although the underlying pathology may influence the appearance of the papilla [78], complicating endoscope insertion, this recent finding had no impact on treatment decisions. The endoscopes were selected depending on the need for the treatment, and anatomical requirements for their use, such as papillotomies, were performed as required.

### 2.6. Data Acquisition

Every patient received a multiple-choice questionnaire after the treatment session and was asked to fill it out on a voluntary basis for each day of the stay until discharge (details are depicted in Table 2). The severity of complaints was examined for each single intervention separately. The results for each requested parameter are given as the frequency of the selected answers (Absolutely all right—Well acceptable—Acceptable—Not acceptable). The questionnaires were collected on discharge and kept until analysis.

The duration of every intervention was recorded. In cases in which the patient received several treatment steps during the same hospital stay (e.g., ISWL and stent insertion on the first day, control SE and stent replacement on the second day), the most invasive procedure was considered as the main procedure, but also other steps were recorded and analyzed as described. Patients whose treatment required more than one separate hospital stay received a questionnaire for each stay, which we considered separately for further analysis.

### 2.7. Data Analysis and Statistics

We used SPSS (Version 26, IBM Corporation, Armonk, NY, USA) for statistical analysis. Bivariate correlations between variables were calculated using Spearman’s correlation coefficient. For the calculation of significances when comparing different groups, we used the Chi-Square test for categorical values, and the Mann–Whitney-U test/Kruskal–Wallis-ANOVA test for continuous values. *p*-values < 0.05 were considered significant.

## 3. Results

### 3.1. Study Population

In total, 377 patients (218 women, 159 men; mean age 52.2 ± 15.0 years, range 13–95 years) agreed to be included in our study. During the study time, we analyzed a total of 470 hospital stays at our department (mean: 1.25 per patient, range 1–6 stays; duration 1–3 days). In 312 patients (312/377, 82.8%), only one stay was necessary until the end of the study time; in 65 patients (65/377, 17.2%), the entire treatment required more than one hospital stay.

### 3.2. Treatments and Admission of Patients

All interventions scheduled for LA were conducted either by the first or the senior author of the study and were successfully performed under LA (N = 470, 100%). No complications occurred during the interventions. All therapeutic goals could be achieved in these treatment sessions, except in one patient (1/377, 0.27%) who interrupted the procedure of a diagnostic SE under LA due to anxiety, not because of pain. Diagnostic SE under GA was performed subsequently without complications.

Details about the interventions are illustrated in Table 3. In a total of 470 interventions, we treated 265 parotid glands (PG; 104 right side, 115 left side, 23 bilateral), 227 submandibular glands (SMG; 124 right side, 101 left side, 1 bilateral), and 3 sublingual glands due to a ranula (SLG; 3 left side; 2 marsupialisations, 1 sublingual gland extirpation). The mean grade of invasiveness was 2.49 ± 1.31 (min. 1, max. 4). We performed 170 procedures with an invasiveness grade of 1 (36.2%), as well as 69 (14.7%), 60 (12.8%), and 171 (36.4%) with grade 2, 3, and 4, respectively (Table 3).

262 of 470 (55.7%) were primary interventions, 208 of 470 (44.3%) were subsequent interventions, either after prior treatment(s) in our department or as a revision treatment after prior therapy elsewhere.

### 3.3. Patients’ Subjective Treatment Judgment

We evaluated 470 questionnaires for 470 hospital stays (100%), considering the most invasive intervention during each stay, in 377 patients.

**Figure 2:**
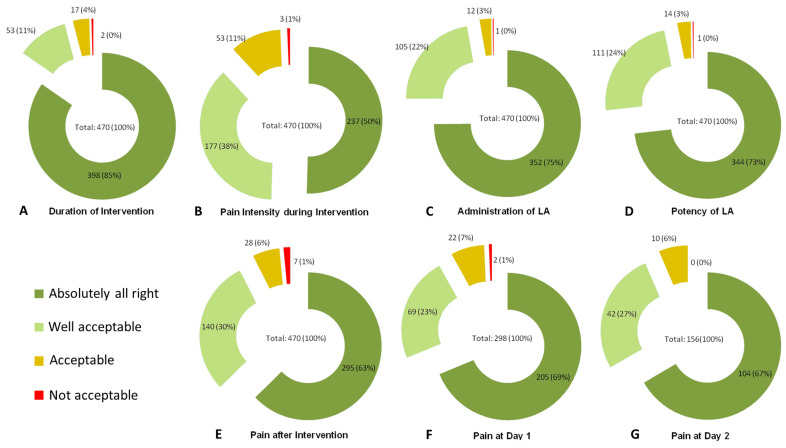
Patient evaluations of individual intervention steps.

The mean duration of an intervention was 30 ± 20 min (range: 5–133 min). In 398 of 470 answers (84.7%), it was explained that the duration was “absolutely all right”, in 53 answers (11.3%) it was “well acceptable”, both adding up to 96% (Figure 2A).

In 237 cases (237/470, 50.4%), the patients evaluated the pain intensity during the intervention as “absolutely all right”, and in 177 cases (37.7%) as “well acceptable”, both adding up to 88.1% in total (Figure 2B).

The administration of the local anesthetic by instillation into the duct and/or injection into the mucosa/tissue was considered “absolutely all right” in 352/470 (74.9%) or “well acceptable” in 105/470 (22.3%) procedures, totaling more than 97% (Figure 2C).

In 344 and 111 of 470 interventions (73.2% and 23.6%, respectively), taken together in 97%, the local anesthetic potency was rated as “absolutely all right” or “well acceptable” (Figure 2D).

The pain intensity directly after the intervention was “absolutely all right” or “well acceptable” in 295 (62.8%) and 140 (29.8%) cases, adding up to 93% in total (Figure 2E).

As the duration of the patients’ hospital stay was related to the intervention performed (Table 1), information about pain intensity on day one and day two was only available from patients who still received inpatient treatment at that time (day one: 298 or 63.4%; day two: 156 or 33.2%, respectively). In total, 92% of the patients on day one and 94% of the patients on day two explained that pain was “absolutely all right” or “well acceptable” (Figure 2F,G).

The question of whether the patients would have the procedure carried out again was answered in all cases (N = 470, 100%). After 470 interventions, 453 (96.4%) patients said they would have the intervention repeated under LA in the same way, while 13 (2.8%) patients answered that they would have the treatment repeated but would prefer the intervention under GA or deeper sedation (Table 4). Only two patients (0.43%) stated that they would not have the treatment repeated: the first because of a temporary facial nerve paresis after instillation of the local anesthetic into the parotid duct (Table 4,^a^), the second due to a dissatisfying combination of pain and duration during an ISWL (Table 4,^c^).

### 3.4. Statistical Analysis of Different Parameters of the Interventions

In 96%, 88%, 97%, and 97%, the duration of the (main) procedure, the pain during the procedure, the administration of LA, and the potency of LA were rated as at least “well acceptable” or better, respectively (Figure 2A–D).

Despite this high acceptance in total, it became obvious that the patients’ acceptance for some investigated parameters decreased with increasing levels of invasiveness. When we compared different levels of invasiveness, significant differences were noted in the patients’ answers regarding “administration of LA” (*p* = 0.006), “duration of the main procedure” (*p* = 0.001), and “pain after the procedure” (*p* = 0.004). For the answers regarding “pain intensity during the intervention”, we did not observe statistically significant differences, although the *p*-values approached the region of significance (*p* = 0.059) with increasing levels of invasiveness. On the contrary, we detected no significant different answers regarding “potency of LA” (*p* = 0.15).

The grade of invasiveness was significantly associated and positively correlated with several parameters (Table 5): the objective duration of the main intervention and the sum of all objective durations (if several interventions were performed in one stay), with the total number of procedures per stay (Table 5, lines 1–3), with the subjective main intervention’s duration (significant for all glands and for PG interventions, but not for SMG interventions; line 4), and with the pain perceived during the main procedure (significant for PG interventions, close to the significance threshold for all glands, not significant for SMG interventions; line 5). However, we did not find significant associations between the grade of invasiveness and the potency of the administered local anesthetic (Table 5, line 6), supporting the hypothesis that the applied local anesthetic, according to Table 1, was appropriate for every category and regarded as sufficient by the patients.

The patients’ perception of the duration was significantly positively associated with the objective duration of the main procedure and the overall objective duration of all procedures per stay. Notably, the sum of perceived durations, including all procedures per stay and the number of interventions per stay, showed no significant association (Table 5, lines 7–9). The perception of pain was positively associated with the duration of the main procedure and, also, with overall duration of all procedures (if several interventions were performed per stay), indicating that, when compared with shorter interventions, longer procedures resulted in significantly higher pain rating values (Table 5, lines 10–11). The number of procedures per stay was not significantly associated with higher pain rating values (Table 5, line 12).

### 3.5. Statistical Analysis of Parameters in Different Glands

When we compared PGs (N = 242) with SMGs/SLGs (N = 228), the grade of invasiveness, the duration of the main procedure and the duration of all procedures per stay all were significantly higher in SMG patients (*p* = 0.0001 each). The potency of the local anesthetic was rated significantly more effective by SMG patients when compared with PG patients (*p* = 0.009). In parallel, the perception of pain was rated higher by PG patients, although not significant (*p* = 0.060). The patients’ perception of the procedure duration, however, did not differ between the glands.

## 4. Discussion

The treatment of IOS has been characterized by a shift toward minimally invasive modalities over the last three decades. Up to date, the question of which form of anesthesia is useful for which type of procedure has only been addressed in a few publications, and only some of these included patient numbers and the type of anesthesia administered. The results indicate large differences in specialized centers worldwide (Table 6). 

None of the cited reports stratified anesthesiology or pain management according to the invasiveness of the procedures. In general, and independent of the invasiveness of the procedures, after investigating 377 patients with 470 interventions, we found that the duration of the procedure, the pain intensity during the procedure, the administration of LA, and the effectiveness of LA were rated at least “well acceptable” or “absolutely all right” in 97%, 97%, 96%, or 88%, respectively (Figure 2A–D).

Although the overall acceptance was very positive, a more detailed analysis revealed that some parameters were significant and positively associated with increasing levels of invasiveness. The rating of “administration of LA” was assessed significantly differently (*p* = 0.006), which is understandable, since procedures with a higher level of invasiveness also require higher volume and more extensive LA and its application became increasingly unpleasant (Table 1). The “duration of the procedure” increased with the grade of invasiveness, which was perceived accordingly by the patients, with the acceptance differing significantly between the single levels of invasiveness (*p* = 0.0001). Higher levels of “pain after the procedure” (*p* = 0.004) were also significantly positively associated with higher grades of invasiveness. These results are explainable, as the interventions were more complex and lasted longer. However, it is noteworthy that both the subjective assessment of “pain during the procedure” and “potency of LA” (*p* = 0.059 and *p* = 0.15, respectively) were not significantly associated with the levels of invasiveness, thus showing that LA, over all levels of invasiveness, can be considered appropriate. In summary, we concluded from our results that all these interventions, independent from the grade of invasiveness, can be successfully conducted under LA, and a high level of patient acceptance can be achieved if an appropriate anesthesiology or pain management—adopted to the invasiveness of the procedure—is applied (Table 1).

According to our schedule, diagnostic SE and SE-controlled irrigation in patients presenting with sialodochitis both represent interventions with the lowest grade of invasiveness (Table 1). Two publications report on diagnostic SE in adults (±irrigation) either carried out completely under LA [10] or GA [81] (Table 6). Luers et al. reported that during simple SE, mean and maximum systolic and diastolic blood pressures were found to be significantly correlated with the duration of SE. More than 80% of these patients rated the procedure as well tolerable, and at least 90% agreed to have the intervention repeated in the same manner. The authors concluded that SE is well tolerable to be performed under LA; however, GA should be considered in more invasive procedures, complex situations with multiple stones, difficult anatomic preconditions, or a very long expected operation time [80].

The same group analyzed postoperative pain management after diagnostic and non-complex interventional SE, and observed that such procedures were associated with moderate pain levels and can be managed by non-opioid analgesic therapy [86].

CRJP, adult sialodochitis and other autoimmune disease-related IOS were treated by SE-controlled irrigation of the duct system with or without additional intervention in 6–179 cases [7,8,29,33,35,36,37]. Most centers treated children under GA in 91–95% of cases [7,8,37], one study described the treatment under deep sedation [36], and in another study, 88.9% of the children received SE under LA [79]. In two further studies, all adults could be treated under LA [29,35]. Likewise, we indicated non-opioid medication in our regime for procedures with comparable grades of invasiveness 1 and 2 (Table 1).

The form of anesthesia was very heterogeneous during the therapy of duct stenosis: some centers treated all their patients under GA [87], while others—including our center—chose LA in 98–100% of cases [18,49,50]. When classified into our classification of the present study, the level of invasiveness of the interventions performed in these studies ranged between 2 and 4 (Table 1).

Jokela and coauthors described results after performing SE or interventional SE under LA (20%) or LA with sedation (80%) in 89 patients [34]. Neither pre- nor intraoperative anxiety levels correlated with pain, pulse, blood pressure or the type of procedure. In total, 85% and 89% of patients, respectively, rated intraoperative discomfort or pain as mild or absent. In total, 87% of the patients experienced the postoperative pain as mild or absent, and 97% stated that they would agree to another procedure again under the same conditions. However, higher pain levels and discomfort were reported after TDS or a longer operation time. Since cases with interventional SE were also included, the invasiveness of the procedures corresponded to grades 1–3 according to our classification.

In further recent reports, SE under sedation or under monitored anesthesia was compared with SE under GA [82,83,84,88]. Trujillo and coauthors [82] found, independent from the underlying pathology, that procedures in monitored anesthesia had a significantly shorter operation and anesthesiology time, with comparable success rates compared to GA. Notably, significantly more patients (34%) reported pain after GA. It was, therefore, concluded that, in compliant patients, monitored anesthesia could be a reasonable alternative to GA, especially in non-complex pathologies. Bawazeer and coworkers [83] reported that SE success rates were higher with conscious sedation (88.9%) than with GA (79.4%), with complication rates being comparable for both groups. Since all patients considered the intervention under conscious sedation to be well tolerable, the authors concluded that distal, even large, stones can be successfully treated using SE under conscious sedation. Mastrolonardo and coauthors [84] analyzed 172 minimal invasive procedures on PGs and SMGs conducted either under GA or monitored LA. Various pathologies were treated; stones were equally distributed in both groups, with similar sizes and locations. In PG treatments, LA was more often chosen. Compared with GA procedures, monitored LA resulted in significantly shorter hospital stays, shorter duration of anesthesia, and shorter operation time, but similar outcomes in terms of resolution of symptoms, further medical interventions, and complications. The authors stated that monitored LA should be considered for solely endoscopic cases. In another study of this group [88], patients’ subjective perceptions of the previously described procedures were evaluated. Patients’ satisfaction was rated excellent in 77% of GA and in 75% of monitored LA cases. Intraoperative pain was at least tolerable in 72% under monitored LA. While patients who received GA reported that they would favor the same type of anesthesia significantly more often than monitored LA patients (85 vs. 61%), the tolerability of post-operative pain showed a tendency to be better for monitored LA (82 vs. 97%). 

Compared with our data, the procedures in these latter reports [82,83,84,88], performed under monitored anesthesia or conscious sedation, corresponded to a maximum invasiveness of grade 3 according to our categories, with acceptance ratings of these patients worse or comparable.

In TDS, performed in SMG only, several available studies report the usage of LA in 93–100% [17,42,43,85], while others report of GA usage in 96–100% [38,40]. In our classification, levels of invasiveness in TDS range between grade 2 and 4 (papillotomy up to extended duct surgery with submandibulotomy, Table 1). This wide range of invasiveness might explain the significant differences in the form of anesthesia used for TDS in different centers worldwide (Table 6).

This also applies to published results for pneumatic or laser ISWL. Our study group reported treatments under LA (98–100%) [13,55], whereas others in GA (61.3–100%) [44,45,46,47,48], and one center reported treatment in sedation (38.7%) [44]. We classified ISWL as grade 4 in our schedule, primarily due to the usually underlying complex pathological conditions (deep location ± multiple stones) often associated with a longer procedure and higher anesthetic requirements (Table 1).

According to earlier reports, ESWL used to be performed without any anesthesia or sedation in adults [26,61,62,63,64,65,66], while children were commonly treated under GA [62]. Nahlieli et al. described that LA was applied during SE prior to ESWL [68]. As ESWL currently is used in our center at higher energy levels and affects not only the gland itself but also adjacent structures, we rated this modality as grade 4 in our classification and adapted our regimen, as previously published [22].

As our data show, the grade of invasiveness was positively associated with the objective and subjectively perceived duration of the main procedure, the overall objective duration of all procedures per stay, and the number of procedures per stay (Table 5, lines 1–4). The positive correlation between the objective and subjective duration of the interventions shows that the patients generally correctly assess the time required for the interventions (Table 5, lines 8–9). Interestingly, the grade of invasiveness was not significantly positively associated with the perception of pain if calculated for all glands and SMGs, but for PGs (*p* = 0.036), possibly reflecting the more complex innervation in this area (Table 5, line 5). The degree of invasiveness was also not associated with the perceived effectiveness of the local anesthetic (Table 5, line 6). While the rated level of pain was significantly positively correlated with the duration(s) of the main procedure/all procedures, the number of interventions per stay were not (Table 5, lines 10–12). The results underline that a sufficient treatment protocol (Table 1) can mitigate the influence of the procedure’s grade of invasiveness on the patients’ perception of pain. Furthermore, the duration of the planned intervention must be considered accordingly (e.g., by administering additional medication). This seemed to be sufficient in the present study, as 96% of our patients rated the duration of the main procedure as at least “well acceptable” (Figure 2A).

Notably, we found that the mean grade of invasiveness and the mean duration of the procedures were significantly higher in the SMG group when compared with the PG group (*p* = 0.0001 each), reflecting the more unfavorable anatomy of the (more proximal) SMG duct system, as described before [13,14,15,55,71]. On the contrary, the potency of the local anesthetic was rated significantly more effective by SMG patients (*p* = 0.009), while the perception of pain showed a tendency to be higher in PG patients (*p* = 0.060). The results again underline, as discussed above (Table 5, line 5), the more complex and widespread neural supply in the PG.

Overall, 96.4% of all patients stated that they would have the procedure carried out again under LA; in detail, this applies for 97.5% of patients who received interventions of invasiveness grades 1–2, and for 95.2% after invasiveness grades 3–4 (Table 4). Furthermore, nearly all patients rated the following parameters as at least “well acceptable”: duration (96%), perceived pain intensity during (88%) and after the intervention on the same day (93%), one day after (92%), and two days after the procedure (94%; Figure 1).

A limitation of the study is that the data were collected in a single center, which is due to the fact that our department is one of the few high-volume units that carries out the full spectrum of these treatments almost exclusively under LA. Furthermore, the experience of the treating surgeon with regard to the interpretation of the findings as well as the indication and selection of a treatment procedure with its rapid implementation definitely has an influence on the perception and satisfaction of the patients. The described and applied management of the present study is, therefore, no guarantee of the success of the interventions themselves, but clearly shows that successful treatment of this patient population is possible under appropriate and optimized conditions.

## 5. Conclusions

To present, existing studies (Table 6) have not provided comparable and comprehensive data on the intra- and perioperative management of IOS. Although a few reports exist that conclude that procedures with a “lower level of invasiveness” can be successfully performed under LA or sedation, procedures with a higher grade of invasiveness are almost exclusively performed under GA at almost all centers worldwide [34,80,82,83,86]. Our results now show that minimally invasive procedures in IOS patients can be performed successfully under LA with high patient acceptance across all grades of invasiveness when comprehensive routine local anesthesia and pain management is applied. As the patients’ acceptance was almost equally distributed across all groups, we conclude that the proposed anesthetic and pain management is sufficient. This makes the treating physician independent of an anesthesiologist, shortens treatment times, saves human and material resources, and creates capacity for operations that must necessarily be carried out under GA.

## Figures and Tables

**Table 1:** Pre-, intra- and postoperative treatment regime, adapted to the invasiveness of the procedure.

			Preoperative	Intraoperative Local Anesthetic ^Ω^			Postoperative			
Grade of Invasiveness	Specific Intervention	Diagnosis or Indication	Oral Premedication	Superficial Mucosa Anesthesia	Intraductal Anesthesia by Instillation into the Duct	Local Anesthesia by Injection into the Mucosa	Antibiotic Treatment	Corticoid	Further Medication	Discharge from Inpatient Treatment
1 (very low)	SE ICI	control sialendoscopy; sialodochitis; mucous plugs; exclusion of stone or stenosis	None	Lidocaine spray (10 mg/hub)—2 hubs	2% articaine, 0.006 mg/mL epinephrine—2–3 mL	2% articaine, 0.006 mg/mL epinephrine—2–3 mL	None	None	2 × 500 mg oral naproxen for 2–3 days 1 × 40 mg oral pantoprazol for 2–3 days	Same day
2 (low)	SSE with papillotomy * Papillotomy * Distal Duct Surgery * Stent Removal † Stent Replacement †	Papillary stenosis (SMG), distal duct stenosis (SMG), distal stone (SMG); small stone or fragment removal with basket or forceps; Stent removal or replacement (PG)	None	Lidocaine spray (10 mg/hub)—2 hubs	2% articaine, 0.006 mg/mL epinephrine—2–5 mL	2% articaine, 0.006 mg/mL epinephrine—3–5 mL	Day 0: 3 g sultamicilline or 600 mg clindamycin intravenously Day 1: 3 × 1.5 g sultamicilline or 4 × 300 mg clindamycin intravenously Day 2: oral sultamicilline or oral clindamycin low dose, for 2–3 days	None	2 × 500 mg oral naproxen for 2–3 days 1 × 40 mg oral pantoprazol for 2–3 days	Day 1 after surgery
3 (medium)	Duct Surgery * Dilation of Stenosis Primary Stent Insertion † Ranula treatment	Duct stenosis (except papilla or distal stenosis in SMG); proximal stones; ranula	50 mg tilidin/4 mg naloxon §	Lidocaine spray (10 mg/hub)—2 hubs	2% articaine, 0.006 mg/mL epinephrine—4–10 mL	2% articaine, 0.006 mg/mL epinephrine—5–10 mL	Day 0: 3 g sultamicilline or 600 mg clindamycin intravenously Day 1–2: 3 × 1.5 g sultamicilline or 4 × 300 mg clindamycin intravenously Day 3: oral sultamicilline or oral clindamycin for 5–6 days	Day 0: Prednisolone 250 mg intravenously (single shot)	2 × 500 mg oral naproxen for 5–6 days 1 × 40 mg oral pantoprazol for 5–6 days	Day 1–2 after surgery
4 (high)	ISWL Extended Duct Surgery * Sublingulectomy ESWL	Hilar stones; complicated stenoses; stones not accessible by endoscopy	50 mg tilidin/4 mg naloxon §	Lidocaine spray (10 mg/hub)—2 hubs	2% articaine, 0.006 mg/mL epinephrine—4–10 mL	2% articaine, 0.006 mg/mL epinephrine ‡—5–10 mL	Day 0: 3 g sultamicilline or 600 mg clindamycin intravenously Day 1–2: 3 × 1.5 g sultamicilline or 4 × 300 mg clindamycin intravenously Day 3: oral sultamicilline or oral clindamycin for 5–6 days	Day 0: Prednisolone 250 mg intravenously (single shot)	2 × 500 mg oral naproxen for 5–6 days 1 × 40 mg oral pantoprazol for 5–6 days	Day 2 after surgery

SE: sialendoscopy; ICI: intraductal corticoid instillation; SSE: sialendoscopy-assisted stone extraction (basket, forceps); ESWL: extracorporeal shock wave lithotripsy; ISWL: intraductal endoscopic shock wave lithotripsy (laser or pneumatic); SMG: submandibular gland; PG: parotid gland. * Papillotomies and duct surgery were performed on the submandibular gland (SMG) only. Distal duct surgery included the distal third of the submandibular duct. Duct surgery (not otherwise specified) included the entire length of the submandibular duct up to the hilum. Extended duct surgery included the entire length of the submandibular duct with a transoral submandibulotomy and creation of a neo-ostium in the posterior floor of the mouth. † Stents were used for parotid gland treatments only in patients treated in this study. § 50 mg tilidinhydrochloride, including 4 mg naloxonhydrochloride. Ω When 2% articaine hydrochloride/0.006 mg/mL epinephrine were applied by instillation and injection, the maximum daily dose was respected in each case (0.175 mL/kg body weight). ‡ If ESWL was performed, 5 mL of 2% articaine hydrochloride/0.006 mg/mL epinephrine was injected into the stone bed and adjacent tissue transorally, and 2–4 mL was additionally injected transcutaneously directly into the stone bed under US control.

**Table 2:** Questionnaire on patients’ perceptions.

Question	Selectable Answers
General questions about the procedure: How did you perceive…	
…the duration of the intervention?	**Categorical scale** Absolutely all right Well acceptable Acceptable Not acceptable
…the pain intensity during the intervention?	
…the application of the local anesthetic (instillation into duct and/or injection)?	
…the potency of your local anesthesia?	
After the procedure: How did you perceive the pain or discomfort…	
…directly after the intervention?	
…on the first day after the intervention?	
…on the second day after the intervention?	
Final assessment: please assess the procedure as a whole…	
I would have the procedure carried out again in the same way:	Yes, also under local anesthesia Yes, but only under general anesthesia No
If you would not have the procedure carried out again in this form: why?	Individual comment of the patient

**Table 3:** Interventions performed under LA in detail.

Treatmnet Sessions and Interventions	N	Gender (F:M)	Grade of Invasiveness
Sialendoscopy only *	135	79:56	1
Sialendoscopy * + cortisone instillation	35	29:6	1
ESWL	25	8:17	4
TDS distal, papillotomy	44	31:13	2
TDS	14	9:5	3
TDS extended with SMT	61	30:31	4
ISWL	46	17:29	4
ISWL + stent	20	10:10	4
Stent insertion	27	20:7	3
Stent removal	24	13:11	2
Dilation of stenosis	17	11:6	3
Dilation of stenosis + stent	13	11:2	4
SE+ Ranula marsupialisation	2	1:1	3
**Other**			
SE + extirpation of SLG	1	0:1	4
ISWL and ESWL ± stent insertion	2	1:1	4
Excision of buccal suture granuloma + SE with stent insertion	1	1:0	2
Dilation of stenosis, stent insertion, ESWL	2	1:1	4
Stent insertion, ESWL	1	0:1	4
**Total**	**470**	**272:198**	

Every patient received at least one diagnostic sialendoscopy. * Sialendoscopy with and without the usage of basket, drill, forceps, but without other interventions. N: Number of treatments performed; ESWL: Extracorporeal Shock Wave Lithotripsy; TDS: Transoral duct surgery; SMT: Submandibulotomy; ISWL: Intracorporeal/Intraductal Shock Wave Lithotripsy (laser or pneumatic); SE: Sialendoscopy; SLG: Sublingual gland.

**Table 4:** Would patients have the procedure carried out again?

	Grade of Invasiveness	Number of Interventions (N, %)	Yes, Under LA	Yes, but Under GA	No	Other (Comment)
	1	168 (35.7%)	165	1	1 ^a^	1 ^b^
	2	71 (15.1%)	68	3	0	0
	3	60 (12.8%)	59	1	0	0
	4	171 (36.4%)	161	8	1 ^c^	1 ^d^
**Total**		**470 (100%)**	**453 (96.4%)**	**13 (2.8%)**	**2 (0.43%)**	**2 (0.43%)**

^a^: Facial nerve paresis due to instillation of LA into the parotid duct, which vanished completely 90 min after sialendoscopy; no further complaints. ^b^: Sedation preferred due to nervousness. ^c^: ISWL intervention: combination of pain (answer: “acceptable”) and duration (82 min) “dissatisfying”. The patient was finally stone free and symptom-free after the intervention and in the follow-up examination eight weeks later. ^d^: Complaints while having the stone were lower than the complaints during the intervention (extended duct surgery with submandibulotomy, SMG), sedation preferred.

**Table 5:** Statistical analysis of different parameters and their associations and correlations.

Parameters Analyzed (Association, Correlation)		All Glands (n = 470)	PGs Only (n = 242)	SMGs Only (n = 225)
Grade of Invasiveness…				
1	and objective duration of main procedure	*p* = 0.0001 *; 0.718, *p* = 0.0001 ^+^	*p* = 0.0001 *; 0.638, *p* = 0.0001 ^+^	*p* = 0.0001 *; 0.715, *p* = 0.0001 ^+^
2	and overall objective durations (sum of durations of all procedures per stay)	*p* = 0.0001 *; 0.736, *p* = 0.0001 ^+^	*p* = 0.0001 *; 0.678, *p* = 0.0001 ^+^	*p* = 0.0001 *; 0.729, *p* = 0.0001 ^+^
3	and number of all procedures per stay	*p* = 0.0001 ^#^	*p* = 0.0001 ^#^	*p* = 0.0001 ^#^
4	and perception of duration of main procedure	*p* = 0.001 ^#^	*p* = 0.05 ^#^	n.s. (*p* = 0.091) ^#^
5	and perception of pain during the main procedure	n.s. (*p* = 0.059) ^#^	*p* = 0.036 ^#^	n.s. ^#^
6	of main procedure and perception of potency of local anesthetic	n.s. ^#^	n.s. ^#^	n.s. ^#^
Perceived duration…				
7	(overall duration—all procedures per stay) and number of procedures per stay	n.s. ^#^	n.s. ^#^	n.s. (*p* = 0.092) ^#^
8	and objective duration of main procedure	*p* = 0.0001 *	*p* = 0.001 *	*p* = 0.003 *
9	and overall objective duration (sum of durations of all procedures per stay)	*p* = 0.0001 *	*p* = 0.002 *	*p* = 0.003 *
Pain …				
10	and duration of main procedure	*p* = 0.0001 *	*p* = 0.0001 *	*p* = 0.01 *
11	and overall duration (sum of durations of all procedures per stay)	*p* = 0.0001 *	*p* = 0.0001 *	*p* = 0.005 *
12	and number of procedures per stay	n.s. ^#^	n.s. ^#^	n.s. ^#^

# Chi-Square Test/Chi-Square Exact Test. * Kruskal–Wallis-ANOVA Test. + Correlation-coefficient according to Spearman.

**Table 6:** Studies about IOS treatment including patient numbers and type of anesthesia.

Intervention	Author, Year	PMID	Number of Patients/ Procedures *	% LA	% GA	Comment
SE ± Cortisone	Konstantinidis I et al., 2011 [79]	21131065	9	88.9	11.1	Children
SE ± Cortisone	Canzi P et al., 2013 [7]	24376291	179	5	95	Review; children
SE ± Cortisone	Ramakrishna J et al., 2014 [8]	25393103	120	10.8	89.2	Review; children
SE ± Cortisone	Capaccio P et al., 2016 [35]	27200511	54	100	0	---
SE ± Cortisone	Capaccio P et al., 2018 [29]	28585263	22	100	0	---
SE ± Cortisone	Capaccio P et al., 2021 [36]	33451162	6	0	100	Children, 100% Sedation
SE ± Cortisone	Geisthoff UW et al., 2022 [33]	34117898	6	0	0	Children; no anesthesia
						
SE ± Intervention	Luers J et al., 2012 [80]	22606931	84	100	0	---
SE ± Intervention	Vashisshta R et al., 2013 [81]	23712592	258	0	100	---
SE ± Intervention	Jokela J et al., 2017 [34]	27659498	89	100	0	80% in sedation
SE ± Intervention	Trujillo et al., 2017 [82]	28520832	65/70 *	38.6 *	61.4 *	38.6% under monitored anesthesia
SE ± Intervention	Bawazeer et al., 2018 [83]	30220479	70	51.4	48.6	51.4% in sedation
SE ± Intervention	Kanerva et al., 2020 [37]	31972384	42	9	91	Children
SE ± Intervention	Mastrolonardo E et al., 2021 [84]	33125904	172	43	57	43% under monitored anesthesia
						
SE and ISWL (Laser)	Durbec m et al., 2012 [44]	23224989	63	0	61.3	38.7% in sedation
SE and ISWL (Laser)	Martellucci et al., 2013 [45]	23462654	16	0	100	---
SE and ISWL (Laser)	Phillips J et al., 2014 [46]	24598407	31	0	100	---
SE and ISWL (Laser)	Sun YT et al., 2014 [47]	25216563	1 (case report)	0	100	---
SE and ISWL (Pneumatic)	Koch M et al., 2016 [55]	26845571	44	100	0	---
SE and ISWL (Pneumatic)	Koch M et al., 2019 [13]	30296893	39	100	0	---
SE and ISWL (Laser and Pneumatic)	Ozdemir et al., 2020 [48]	32569053	51	0	100	---
SE and ISWL (Laser)	Koch et al., 2021 [14]	32997838	49	98	2	---
SE and ISWL (Pneumatic)	Koch M et al., 2022 [15]	34637368	62	100	0	---
						
TDS SMG	McGurk M et al., 2004 [38]	15337182	55	3.6	96.4	---
TDS SMG	Zenk J et al., 2005 [42]	15184990	638	99	1	---
TDS SMG	Capaccio P et al., 2011 [85]	21298387	84	100	0	---
TDS SMG	Liu DG et al., 2013 [43]	22520565	70	93	7	---
TDS SMG	Schapher M et al., 2017 [17]	28052363	234	98	2	---
TDS SMG	Shi H et al., 2020 [40]	32444816	9	0	100	---
						
ESWL	Iro H et al., 1998 [61]	9793530	76	99	1	---
ESWL	Ottaviani F et al., 2001 [62]	11718255	7	0	1 Sedation	No anesthesia
ESWL	Escudier MP et al., 2003 [63]	12673752	122	0	0	No anesthesia
ESWL	Zenk J et al., 2004 [64]	15174765	197	0	0	No anesthesia
ESWL	Capaccio P et al., 2004 [65]	15179215	322	0	0	No anesthesia
ESWL	Guerre A et al., 2011 [66]	21345475	1571	0	0	No anesthesia

IOS: Inflammatory and Obstructive Sialadenitis; SE: Sialendoscopy; TDS: Transoral Duct Surgery; SMG: Submandibular Gland; ISWL: intraductal/intracorporeal shock wave lithotripsy; ESWL: Extracorporeal Shock Wave Lithotripsy. * Number of procedures.

## Data Availability

The data supporting the reported results can be requested from the authors.

## References

[1] Capaccio P., Gaffuri M., Rossi V., Pignataro L. (2017). Sialendoscope-assisted transoral removal of hilo-parenchymal sub-mandibular stones: Surgical results and subjective scores. Acta Otorhinolaryngol. Ital..

[2] Koch M., Zenk J., Iro H. (2009). Algorithms for treatment of salivary gland obstructions. Otolaryngol. Clin. N. Am..

[3] Koch M., Mantsopoulos K., Muller S., Sievert M., Iro H. (2021). Treatment of Sialolithiasis: What Has Changed? An Update of the Treatment Algorithms and a Review of the Literature. J. Clin. Med..

[4] Sigismund P.E., Zenk J., Koch M., Schapher M., Rudes M., Iro H. (2015). Nearly 3000 salivary stones: Some clinical and epidemiologic aspects. Laryngoscope.

[5] Koch M., Iro H. (2017). Salivary duct stenosis: Diagnosis and treatment. Acta Otorhinolaryngol. Ital..

[6] Capaccio P., Sigismund P.E., Luca N., Marchisio P., Pignataro L. (2012). Modern management of juvenile recurrent parotitis. J. Laryngol. Otol..

[7] Canzi P., Occhini A., Pagella F., Marchal F., Benazzo M. (2013). Sialendoscopy in juvenile recurrent parotitis: A review of the literature. Acta Otorhinolaryngol. Ital..

[8] Ramakrishna J., Strychowsky J., Gupta M., Sommer D.D. (2015). Sialendoscopy for the management of juvenile recurrent parotitis: A systematic review and meta-analysis. Laryngoscope.

[9] Marchal F., Dulguerov P., Lehmann W. (1999). Interventional sialendoscopy. N. Engl. J. Med..

[10] Koch M., Zenk J., Bozzato A., Bumm K., Iro H. (2005). Sialoscopy in cases of unclear swelling of the major salivary glands. Otolaryngol Head Neck Surg.

[11] Strychowsky J.E., Sommer D.D., Gupta M.K., Cohen N., Nahlieli O. (2012). Sialendoscopy for the management of obstructive salivary gland disease: A systematic review and meta-analysis. Arch. Otolaryngol. Head Neck Surg..

[12] Witt R.L., Iro H., Koch M., McGurk M., Nahlieli O., Zenk J. (2012). Minimally invasive options for salivary calculi. Laryngoscope.

[13] Koch M., Schapher M., Mantsopoulos K., Goncalves M., Iro H. (2019). Intraductal Pneumatic Lithotripsy after Extended Transoral Duct Surgery in Submandibular Sialolithiasis. Otolaryngol. Head Neck Surg..

[14] Koch M., Schapher M., Mantsopoulos K., Iro H. (2021). Intraductal Lithotripsy in Sialolithiasis Using the Calculase III Ho:YAG Laser: First Experiences. Lasers Surg. Med..

[15] Koch M., Schapher M., Sievert M., Mantsopoulos K., Iro H. (2021). Intraductal Fragmentation in Sialolithiasis Using Pneumatic Lithotripsy: Initial Experience and Results. Otolaryngol. Head Neck Surg..

[16] Schapher M., Goncalves M., Mantsopoulos K., Iro H., Koch M. (2020). Papillary stenosis of the submandibular gland caused by dental prostheses. Oral. Surg. Oral. Med. Oral. Pathol. Oral. Radiol..

[17] Schapher M., Mantsopoulos K., Messbacher M.E., Iro H., Koch M. (2017). Transoral submandibulotomy for deep hilar submandibular gland sialolithiasis. Laryngoscope.

[18] Koch M., Iro H., Zenk J. (2008). Role of sialoscopy in the treatment of Stensen’s duct strictures. Ann. Otol. Rhinol. Laryngol..

[19] Koch M., Kunzel J., Iro H., Psychogios G., Zenk J. (2014). Long-term results and subjective outcome after gland-preserving treatment in parotid duct stenosis. Laryngoscope.

[20] Ye X., Zhang Y.Q., Xie X.Y., Liu D.G., Zhang L., Yu G.Y. (2017). Transoral and transcutaneous approach for removal of parotid gland calculi: A 10-year endoscopic experience. Oral. Surg. Oral. Med. Oral. Pathol. Oral. Radiol..

[21] Koch M., Iro H., Zenk J. (2013). Combined endoscopic-transcutaneous surgery in parotid gland sialolithiasis and other ductal diseases: Reporting medium- to long-term objective and patients’ subjective outcomes. Eur. Arch. Otorhinolaryngol..

[22] Koch M., Schapher M., Mantsopoulos K., von Scotti F., Goncalves M., Iro H. (2018). Multimodal treatment in difficult sialolithiasis: Role of extracorporeal shock-wave lithotripsy and intraductal pneumatic lithotripsy. Laryngoscope.

[23] Capaccio P., Torretta S., Pignataro L., Koch M. (2017). Salivary lithotripsy in the era of sialendoscopy. Acta Otorhinolaryngol. Ital..

[24] Gallo A., Capaccio P., Benazzo M., De Campora L., De Vincentiis M., Farneti P., Fusconi M., Gaffuri M., Lo Russo F., Martellucci S. (2016). Outcomes of interventional sialendoscopy for obstructive salivary gland disorders: An Italian multicentre study. Acta Otorhinolaryngol. Ital..

[25] Marchal F., Dulguerov P. (2003). Sialolithiasis management: The state of the art. Arch Otolaryngol. Head Neck Surg..

[26] Iro H., Zenk J., Escudier M.P., Nahlieli O., Capaccio P., Katz P., Brown J., McGurk M. (2009). Outcome of minimally invasive management of salivary calculi in 4691 patients. Laryngoscope.

[27] Foletti J.M., Graillon N., Avignon S., Guyot L., Chossegros C. (2018). Salivary Calculi Removal by Minimally Invasive Techniques: A Decision Tree Based on the Diameter of the Calculi and Their Position in the Excretory Duct. J. Oral. Maxillofac. Surg..

[28] Lommen J., Schorn L., Roth B., Naujoks C., Handschel J., Holtmann H., Kubler N.R., Sproll C. (2021). Sialolithiasis: Retrospective analysis of the effect of an escalating treatment algorithm on patient-perceived health-related quality of life. Head Face Med..

[29] Capaccio P., Canzi P., Torretta S., Rossi V., Benazzo M., Bossi A., Vitali C., Cavagna L., Pignataro L. (2018). Combined interventional sialendoscopy and intraductal steroid therapy for recurrent sialadenitis in Sjogren’s syndrome: Results of a pilot monocentric trial. Clin. Otolaryngol..

[30] Familiari M., Ferrario F., Giordano L., Santo D.D., Indelicato P., Battista R.A., Bussi M. (2021). Management of obstructive pathology of the salivary glands in elderly patients: A preliminary study. J. Laryngol. Otol..

[31] Hardcastle T., Rasul U., de Paiva Leite S., Zheng K., Donaldson G., Ahmad Z., Morton R.P. (2021). The Manukau Salivary Symptoms Score for Assessing the Impact of Sialendoscopy in Recurrent Obstructive Sialadenitis. Otolaryngol. Head Neck Surg..

[32] Iro H., Zenk J., Koch M. (2010). Modern concepts for the diagnosis and therapy of sialolithiasis. HNO.

[33] Geisthoff U.W., Droege F., Schulze C., Birk R., Rudhart S., Maune S., Stuck B.A., Hoch S. (2021). Treatment of juvenile recurrent parotitis with irrigation therapy without anesthesia. Eur. Arch. Otorhinolaryngol..

[34] Jokela J., Haapaniemi A., Makitie A., Saarinen R. (2017). Sialendoscopy under local anaesthesia. Acta Otolaryngol..

[35] Capaccio P., Torretta S., Di Pasquale D., Rossi V., Pignataro L. (2017). The role of interventional sialendoscopy and intraductal steroid therapy in patients with recurrent sine causa sialadenitis: A prospective cross-sectional study. Clin. Otolaryngol..

[36] Capaccio P., Palermo A., Lucchinelli P., Marchesi T., Torretta S., Gaffuri M., Marchisio P., Pignataro L. (2021). Deep Sedation for Pediatric Parotid Sialendoscopy in Juvenile Recurrent Parotitis. J. Clin. Med..

[37] Kanerva M., Tapiovaara L., Aro K., Saarinen R. (2020). Pediatric sialendoscopy: An 11-year study from a single tertiary care center. Int. J. Pediatr. Otorhinolaryngol..

[38] McGurk M., Makdissi J., Brown J.E. (2004). Intra-oral removal of stones from the hilum of the submandibular gland: Report of technique and morbidity. Int. J. Oral. Maxillofac. Surg..

[39] Eun Y.G., Chung D.H., Kwon K.H. (2010). Advantages of intraoral removal over submandibular gland resection for proximal submandibular stones: A prospective randomized study. Laryngoscope.

[40] Shi H., Zhao J., Hze-Khoong E.P., Liu S., Yin X., Hu Y. (2020). A gland-sparing, intraoral sialolithotomy approach for hilar and intraparenchymal multiple stones in the submandibular gland. Sci. Rep..

[41] Sproll C., Naujoks C., Holtmann H., Kubler N.R., Singh D.D., Rana M., Lommen J. (2019). Removal of stones from the superficial lobe of the submandibular gland (SMG) via an intraoral endoscopy-assisted sialolithotomy. Clin. Oral. Investig..

[42] Zenk J., Gottwald F., Bozzato A., Iro H. (2005). Submandibular sialoliths. Stone removal with organ preservation. HNO.

[43] Liu D.G., Jiang L., Xie X.Y., Zhang Z.Y., Zhang L., Yu G.Y. (2013). Sialoendoscopy-assisted sialolithectomy for submandibular hilar calculi. J. Oral. Maxillofac. Surg..

[44] Durbec M., Dinkel E., Vigier S., Disant F., Marchal F., Faure F. (2012). Thulium-YAG laser sialendoscopy for parotid and submandibular sialolithiasis. Lasers Surg. Med..

[45] Martellucci S., Pagliuca G., de Vincentiis M., Greco A., Fusconi M., De Virgilio A., Gallipoli C., Gallo A. (2013). Ho:Yag laser for sialolithiasis of Wharton’s duct. Otolaryngol. Head Neck Surg..

[46] Phillips J., Withrow K. (2014). Outcomes of Holmium Laser-Assisted Lithotripsy with Sialendoscopy in Treatment of Sialolithiasis. Otolaryngol. Head Neck Surg..

[47] Sun Y.T., Lee K.S., Hung S.H., Su C.H. (2014). Sialendoscopy with holmium:YAG laser treatment for multiple large sialolithiases of the Wharton duct: A case report and literature review. J. Oral. Maxillofac. Surg..

[48] Ozdemir S. (2020). Outcomes of Pneumatic Lithotripsy Versus Holmium Laser-Assisted Lithotripsy With Sialendoscopy in Management of Submandibular Sialolithiasis. J. Craniofac. Surg..

[49] Koch M., Iro H., Kunzel J., Psychogios G., Bozzato A., Zenk J. (2012). Diagnosis and gland-preserving minimally invasive therapy for Wharton’s duct stenoses. Laryngoscope.

[50] Koch M., Zenk J., Iro H. (2008). Diagnostic and interventional sialoscopy in obstructive diseases of the salivary glands. HNO.

[51] Marchal F., Dulguerov P., Becker M., Barki G., Disant F., Lehmann W. (2001). Specificity of parotid sialendoscopy. Laryngoscope.

[52] Marchal F., Dulguerov P., Becker M., Barki G., Disant F., Lehmann W. (2002). Submandibular diagnostic and interventional sialendoscopy: New procedure for ductal disorders. Ann. Otol. Rhinol. Laryngol..

[53] Nahlieli O., Shacham R., Bar T., Eliav E. (2003). Endoscopic mechanical retrieval of sialoliths. Oral. Surg. Oral. Med. Oral. Pathol. Oral. Radiol. Endodontology.

[54] Zenk J., Koch M., Klintworth N., Konig B., Konz K., Gillespie M.B., Iro H. (2012). Sialendoscopy in the diagnosis and treatment of sialolithiasis: A study on more than 1000 patients. Otolaryngol. Head Neck Surg..

[55] Koch M., Mantsopoulos K., Schapher M., von Scotti F., Iro H. (2016). Intraductal pneumatic lithotripsy for salivary stones with the StoneBreaker: Preliminary experience. Laryngoscope.

[56] Samani M., Hills A.J., Holden A.M., Man C.B., McGurk M. (2016). Minimally-invasive surgery in the management of symptomatic parotid stones. Br. J. Oral. Maxillofac. Surg..

[57] Carroll W.W., Walvekar R.R., Gillespie M.B. (2013). Transfacial ultrasound-guided gland-preserving resection of parotid sialoliths. Otolaryngol. Head Neck Surg..

[58] Joshi A.S., Sood A.J. (2014). Ultrasound-Guided Needle Localization during Open Parotid Sialolithotomy. Otolaryngol. Head Neck Surg..

[59] Nahlieli O., London D., Zagury A., Eliav E. (2002). Combined approach to impacted parotid stones. J. Oral. Maxillofac. Surg..

[60] Zheng L., Xie L., Wang Z., Shi H., Cao N., Yu C. (2015). Endoscopic-assisted gland preserving therapy for the management of parotid gland sialolithiasis: Our preliminary experience. J. Craniomaxillofac. Surg..

[61] Iro H., Zenk J., Hornung J., Schneider T., Ell C. (1998). Long-term results of extracorporeal peizoelectric shock wave lithotripsy of parotid stones. Dtsch. Med. Wochenschr..

[62] Ottaviani F., Marchisio P., Arisi E., Capaccio P. (2001). Extracorporeal shockwave lithotripsy for salivary calculi in pediatric patients. Acta Otolaryngol..

[63] Escudier M.P., Brown J.E., Drage N.A., McGurk M. (2003). Extracorporeal shockwave lithotripsy in the management of salivary calculi. Br. J. Surg..

[64] Zenk J., Bozzato A., Winter M., Gottwald F., Iro H. (2004). Extracorporeal shock wave lithotripsy of submandibular stones: Evaluation after 10 years. Ann. Otol. Rhinol. Laryngol..

[65] Capaccio P., Ottaviani F., Manzo R., Schindler A., Cesana B. (2004). Extracorporeal lithotripsy for salivary calculi: A long-term clinical experience. Laryngoscope.

[66] Guerre A., Katz P. (2011). Extracorporeal shockwave lithotripsy (ESWL) for salivary gland stones: A retrospective study of 1571 patients. Rev. Stomatol. Chir. Maxillofac..

[67] Katz P. (2004). New techniques for the treatment of salivary lithiasis: Sialoendoscopy and extracorporal lithotripsy: 1773 cases. Ann. Otolaryngol. Chir. Cervicofac..

[68] Nahlieli O., Shacham R., Zaguri A. (2010). Combined external lithotripsy and endoscopic techniques for advanced sialolithiasis cases. J. Oral. Maxillofac. Surg..

[69] Goncalves M., Mantsopoulos K., Schapher M., Iro H., Koch M. (2018). Ultrasound Supplemented by Sialendoscopy: Diagnostic Value in Sialolithiasis. Otolaryngol. Head Neck Surg..

[70] Goncalves M., Mantsopoulos K., Schapher M., Iro H., Koch M. (2021). Ultrasound in the diagnosis of parotid duct obstruction not caused by sialolithiasis: Diagnostic value in reference to direct visualization with sialendoscopy. Dentomaxillofac. Radiol..

[71] Koch M., Hung S.H., Su C.H., Lee K.S., Iro H., Mantsopoulos K. (2019). Intraductal lithotripsy in sialolithiasis with two different Ho:YAG lasers: Presetting parameters, effectiveness, success rates. Eur. Rev. Med. Pharmacol. Sci..

[72] Koch M., Schapher M., Goncalves M., Iro H., Mantsopoulos K. (2020). Simultaneous application of ultrasound and sialendoscopy: Experience in the management of stenosis and other non-sialolithiasis-related salivary gland disorders. Eur. Rev. Med. Pharmacol. Sci..

[73] Koch M., Schapher M.L., Mantsopoulos K., Goncalves M., Iro H. (2022). Simultaneous Application of Ultrasound and Sialendoscopy and its Value in the Management of Sialolithiasis. Ultraschall Med..

[74] Badger C.D., Benito D.A., Joshi A.S. (2021). Incorporating Sialendoscopy into the Otolaryngology Clinic. Otolaryngol. Clin. N. Am..

[75] Coniglio A.J., Deal A.M., Bhate O., Hackman T.G. (2019). In-office versus Operating Room Sialendoscopy: Comparison of Outcomes, Patient Time Burden, and Charge Analysis. Otolaryngol. Head Neck Surg..

[76] Mastrolonardo E., Stewart M., Alapati R., Campbell D., Thaler A., Zhan T., Curry J.M., Luginbuhl A.J., Cognetti D.M. (2021). Improved efficiency of sialendoscopy procedures at an ambulatory surgery center. Am. J. Otolaryngol..

[77] Bozzato A., Hertel V., Bumm K., Iro H., Zenk J. (2009). Salivary simulation with ascorbic acid enhances sonographic diagnosis of obstructive sialadenitis. J. Clin. Ultrasound.

[78] Anicin A., Jerman A., Urbancic J., Pusnik L. (2023). Sialendoscopy-Based Analysis of Submandibular Duct Papillae with a Proposal for Classification. J. Clin. Med..

[79] Konstantinidis I., Chatziavramidis A., Tsakiropoulou E., Malliari H., Constantinidis J. (2011). Pediatric sialendoscopy under local anesthesia: Limitations and potentials. Int. J. Pediatr. Otorhinolaryngol..

[80] Luers J.C., Stenner M., Schinke M., Helmstaedter V., Beutner D. (2012). Tolerability of sialendoscopy under local anesthesia. Ann. Otol. Rhinol. Laryngol..

[81] Vashishta R., Gillespie M.B. (2013). Salivary endoscopy for idiopathic chronic sialadenitis. Laryngoscope.

[82] Trujillo O., Drusin M.A., Pagano P.P., Askin G., Rahmati R. (2017). Evaluation of Monitored Anesthesia Care in Sialendoscopy. JAMA Otolaryngol. Head Neck Surg..

[83] Bawazeer N., Carvalho J., Djennaoui I., Charpiot A. (2018). Sialendoscopy under conscious sedation versus general anesthesia. A comparative study. Am. J. Otolaryngol..

[84] Mastrolonardo E., Stewart M., Alapati R., Thaler A., Zhan T., Curry J.M., Luginbuhl A.J., Cognetti D.M. (2021). Comparison of general anesthesia and monitored anesthesia care for sialendoscopy procedures. Am. J. Otolaryngol..

[85] Capaccio P., Clemente I.A., McGurk M., Bossi A., Pignataro L. (2011). Transoral removal of hiloparenchymal submandibular calculi: A long-term clinical experience. Eur. Arch. Otorhinolaryngol..

[86] Nachtsheim L., Pick C., Klussmann J.P., Loser J., Luers J.C. (2019). Analyse und Management von postoperativen Schmerzen nach Sialendoskopie. Laryngorhinootologie.

[87] Papadaki M.E., McCain J.P., Kim K., Katz R.L., Kaban L.B., Troulis M.J. (2008). Interventional sialoendoscopy: Early clinical results. J. Oral. Maxillofac. Surg..

[88] Mastrolonardo E., Campbell D.J., Stewart M., Swendseid B., Thaler A., Curry J.M., Luginbuhl A.J., Cognetti D.M. (2022). Patient experiences of sialendoscopy with monitored anesthesia care versus general anesthesia. Am. J. Otolaryngol..

